
JOURNAL INFORMATION
==============================
NLM Title Abbreviation: ScientificWorldJournal
Journal ID: ScientificWorldJournal
NLM Journal ID: 101131163
Journal ID: TSWJ

The Scientific World Journal

ISSN: 2356-6140
EISSN: 1537-744X
Publisher: Wiley

ARTICLE INFORMATION
==============================
PMCID: PMC6009308
PMID: 29973785
DOI: 10.1100/tsw.2002.11
Article ID: 136727
Article version: 1
Subjects: Short Report

Comparing the Speed and Accuracy of the Smith and Waterman Algorithm as Implemented by Mpsrch with the Blast and Fasta Heuristics for Sequence Similarity Searching

Muratet Michael A. *
ResGen, Invitrogen Corporation, 2130 South Memorial Parkway, Huntsville, Alabama, 35801, USA
*Michael A. Muratet: michael.muratet@invitrogen.com
Publication date: 2002
Electronic publication date: 2002 Apr 15
Volume: 2
First page: 21
Last page: 22
Copyright: Copyright © 2002 Michael A. Muratet.
Copyright year: 2002
License: This is an open access article distributed under the Creative Commons Attribution License, which permits unrestricted use, distribution, and reproduction in any medium, provided the original work is properly cited.
License URL: https://creativecommons.org/licenses/by/3.0/

==============================
Advances in Gene Technology: The Genome and Beyond –
Structural Biology for Medicine (Proceedings of the 2002
Miami *Nature Biotechnology* Winter Symposium)
*TheScientificWorld* 2002, 2(S2), 21–22
ISSN 1532-2246; DOI 10.1100/tsw.2002.11

### COMPARING THE SPEED AND ACCURACY OF THE SMITH AND WATERMAN ALGORITHM AS IMPLEMENTED BY MPSRCH WITH THE BLAST AND FASTA HEURISTICS FOR SEQUENCE SIMILARITY SEARCHING

Michael A. Muratet
ResGen, Invitrogen Corporation, 2130 South Memorial Parkway, Huntsville, Alabama, 35801
michael.muratet@invitrogen.com

**INTRODUCTION.** Similarity searching is used to identify homologies between a query sequence and sequences in a database to elucidate the function of the former by considering the latter. Similarity searching (or more appropriately, dissimilarity searching) is also used in oligomer design, which involves the identification of a unique N-mer (N < 100) to represent a gene for microarray and other assays. The sensitivity of the search is a measure of how well an algorithm can locate all related or matching sequences in the database. The BLAST heuristic is probably the most widely used sequence matching method today due primarily to its availability on public servers with graphical interfaces (such as the one at NCBI) and its speed[1,5]. Many commercial versions are available that are accelerated in some manner. The FASTA heuristic is also used although it is slower than BLAST because it is more sensitive[2,4]. Both of these methods are based on approximations that aggregate the sequence into tokens prior to the search to reduce the computational complexity (i.e., decrease the time to search). The Smith-Waterman algorithm is an exhaustive search based on Bellman's dynamic programming algorithm and is therefore the most sensitive (and historically slowest) of the three methods[3]. In fact, once the approximate methods of BLAST and FASTA have produced sites of potential alignment, it is often the Smith-Waterman that is used to calculate the actual alignment. MPSRCH is an implementation of the Smith-Waterman algorithm that exploits the capabilities of the processor hardware to increase the speed of the algorithm to level similar to BLAST or FASTA. It has been implemented on the Compaq Alpha, the Intel Pentium, and the Motorola PowerPC.

**METHOD.** The sensitivity of these three algorithms is evaluated systematically against a database of proteins or nucleic acids. Each algorithm is tested by selecting one of the genes in the database as the query sequence using the default settings of the algorithm, and by varying the settings to improve the performance.

**RESULTS.** The BLAST algorithm is the least sensitive and occasionally fails to find the query sequence known to be in the database. The FASTA algorithm will also occasionally fail to find matches produced by MPSRCH. Such a failure can be problematic for functional searches, and catastrophic for oligomer design. The results of these tests, the reasons behind the failures, and the implications for users are presented.

**DISCUSSION.** Pearson compared the Smith and Waterman algorithm with FASTA and BLASTP in 1995 and concluded that the Smith and Waterman algorithm and FASTA performed significantly better than BLASTP with modern scoring matrices and that the Smith and Waterman algorithm performed better than FASTA when used with complete sequences[4]. Even so, the computational complexity of the Smith and Waterman algorithm has apparently discouraged its widespread use. Since then, there have been improvements in the BLAST algorithm[5] and new applications for similarity searching have appeared, such as oligomer design for microarrays. Moreover, with recent CPU and memory developments the speed of the MPSRCH implementation of the Smith and Waterman algorithm is approaching that of BLAST. It is therefore valuable to reexamine the sensitivity of these methods in the light of these new developments.

**ACKNOWLEDGEMENTS.** The author is grateful for the assistance of Dr. Shane Sturrock and Dr. Graham Davies of Edinburgh Biocomputing Systems.

### REFERENCES

1. Altschul, S.F., Gish, W., Miller, W., Myers, E.W., and Lipman, D.J. (1990) J. Mol. Biol. 215, 403–410.
2. Pearson, W.R. and Lipman, D.J. (1988) Proc. Natl. Acad. Sci. U. S. A. 85, 2444–2448.
3. Smith, T.F. and Waterman, M. (1981) J. Mol. Biol. 147, 195–197.
4. Pearson, W.R. (1995) Prot. Sci. 4, 1145–1160.
5. Altschul, S.F., Madden, T.L., Schaffer, A.A., Zhang, J., Zhang, Z., Miller, W., and Lipman, D.J. (1997) Nucl. Acids Res. 25, 3389–3402.
